# Private management costs of *Popillia japonica*: a study of viticulture in Italy

**DOI:** 10.3389/finsc.2023.1176405

**Published:** 2023-06-14

**Authors:** Franziska B. Straubinger, Terese E. Venus, Emmanuel O. Benjamin, Johannes Sauer

**Affiliations:** ^1^Chair Group of Production and Resource Economics, Technical University of Munich (TUM), Munich, Germany; ^2^Research Group of Bioeconomy Economics, University of Passau, Passau, Germany

**Keywords:** Japanese beetle, invasive species, biological invasion, pest management, partial budget, grape production, socio-economic impact

## Abstract

The Japanese beetle (*Popillia japonica*) is classified as a high-priority pest in the European Union and is reported to have caused extensive damage to grapevine leaves in Italy. As there are few studies, which measure the beetle’s socio-economic impact, we conduct a first descriptive assessment of grapevine farmers’ perception of the beetle’s impact and assess the pest’s effect on private management costs using a partial budgeting approach. Our sample includes data from 65 producers and 118 vineyard plots. In terms of farmers’ perception, we find that farmers anticipate increased management costs and believe a further spread of the beetle will lead to at least moderate yield and quality damages for the majority of plots (58-91%). While farmers do not expect to stop grapevine cultivation for the majority of vineyard plots, affected farmers they believe it is likely to very likely for 29% of plots. We also find that affected farmers rate their vines’ resilience higher than unaffected farmers do. Using a partial budgeting approach, we find that a Japanese beetle infestation leads on average to a net income decrease of around €2727 per hectare. This decrease is due to an average increase in labor costs of around €1715. Additionally, an average yield reduction that results in a revenue loss of around €966 and additional control costs of around €47 per infested hectare, further contribute to the net income decrease. Even though the small number of observations does not allow us to make conclusions about the beetle’s impact on the Italian viticulture sector as a whole, our findings provide first insights and demonstrate the need for environmentally friendly and effective control products that can replace labor-intensive manual control measures, which are currently applied in Japanese beetle infested vineyards.

## 1 Introduction

**Figure 1:**
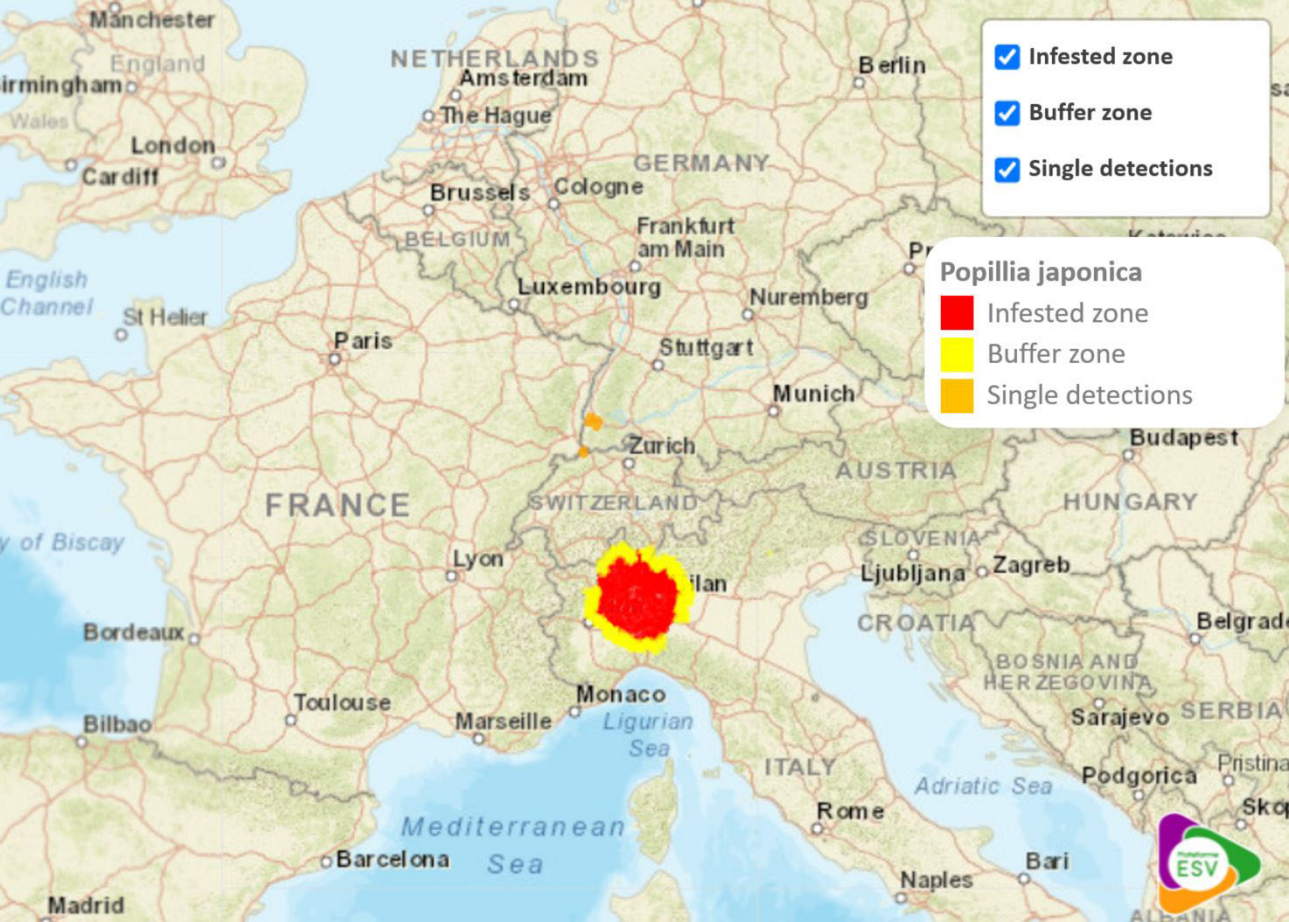
Japanese beetle infestation zone, buffer zone and single detections in mainland Europe. Source: adapted from ESV (13).

The beetle *Popillia japonica* (Japanese beetle), native to Japan, is an invasive insect pest in Europe and considered a major threat to European agriculture. It can feed on more than 300 different host plants (1) and there are many areas in Europe with suitable climatic conditions for its proliferation (2, 3). The beetle is considered a priority pest candidate in the EU (4) and ranked as the 2^nd^ most dangerous pest for crops by the European Food and Safety Authority (5). In mainland Europe, the invasive insect was first detected in Italy in the Ticino Valley Natural Park in 2014 (6). It has since spread rapidly across the country. In 2020, the official infestation zone in Italy covered an area of 7,550 km2 (7) and only two years later in 2022, it increased to 16,232 km2 (Bosio, personal communication)^1^. In Italy, the infested zone covers parts of the Piedmont, Lombardy, and Emilia-Romagna regions, which include major wine-producing areas (8). The delimited area also includes part of the Valle d´Aosta region (9). In addition to Italy, the Japanese beetle has been detected in other European member states including Portugal (Azores) (10), Switzerland (11) and single beetle findings in Germany (12) (**Figure 1**).

Although the Japanese beetle has been in mainland Europe for almost a decade, there are limited studies on its socioeconomic impact on agribusinesses and perception of affected farmers. Until now, the majority of studies have focused on projecting the beetle’s future impact. For example, Straubinger et al. (14) estimate that without effective management of the beetle, a full infestation in the EU could lead to potential annual damage costs of €30 million up to €7.8 billion for the host crops of grain maize, soy, apple, peach, cherry, and grapes. For Italy, it has been estimated that a full Japanese beetle infestation could lead to annual damage costs of around €68 million with grapes accounting for 74% of the damage (14). Grapevine is one of the pests preferred host crops (15, 16) and large numbers of the adult beetle can be found between June and July in infested viticulture areas in Italy (7). Bosio et al. (17) report that vineyards in Piedmont frequently deal with infestations of 200-300 adult Japanese beetles per vine, with peaks of over 1,000 adults. This has prompted concerns about the beetle’s impact on Italian viticulture.

To address these concerns, we assess grape farmers’ perception of the infestation and evaluate the impact of the Japanese beetle on grape yield and private management costs using a partial budgeting approach. Our assessment is based on a survey of 65 Italian grape producers conducted in 2022 as well as secondary data from the literature. We present a descriptive assessment of the pest’s economic damage on yield and use the partial budgeting approach to show the additional costs affected farmers may accrue. To the best of our knowledge, this is the first study assessing the private management costs of the Japanese beetle in Europe using primary data. Thus, despite the small sample size, this study provides an important case study for neighboring regions that may be affected by the beetle in the future.

## 2 Materials and methods

### 2.1 Data compilation

To assess farmer perception and management costs associated with a Japanese beetle infestation in viticulture, we collected quantitative data on production and control methods through an online survey using the survey tool Tivian and combined it with secondary data on labor (18) and material costs (19–22). For the survey, we used a structured questionnaire and voluntary response sampling (see Appendix A). A preliminary version of the questionnaire was designed based on surveys from the existing literature on grapevine cultivation and translated from English to Italian by an Italian native speaker. Before conducting the survey, it was tested by an Italian wine grower, a wine expert and a phytosanitary expert. We developed the final form of the survey incorporating their feedback on the questionnaire draft. The link to access the online survey was distributed through Italy’s largest organization of wine producers (Vignaioli Piemontesi), the phytosanitary service of Piedmont region (Regione Piemonte) and the Council for Agricultural Research and Economics (CREA) from August until October 2022. A minimum of 160 grape producers were invited to participate *via* e-mail through the three institutes. Overall, according to the wine association (Vignaioli Piemontesi), their members produce about 30% of the wine in Piedmont with 325 members, which is made up of 291 individual wineries and 34 cooperative wineries, which represents about 6,000 individual wineries (23). According to the FADN’s public database, in 2020, there were 6,672 wineries in Piedmont and 88,692 in Italy (24). To all participants of the survey, we guaranteed anonymity and the publication of results only in aggregated form. In total, 65 Italian farmers completed the survey and provided information on 119 vineyard plots.

The survey included questions about farm characteristics and management practices that could be affected by a Japanese beetle infestation in 2021 (see **Table 1** for an overview of potential additional management costs). The data were collected on the plot level to be able to compare affected and unaffected plots within the same farm. In addition, to understand the farmers’ perception of the Japanese beetle’s future impact on vineyards, we included questions about how a spread of the pest will affect the yield, quality, management costs and future cultivation of their vineyard plots. Moreover, the survey contained questions about how farmers perceive the leaf damage tolerance level of their vines. We considered this question important as the beetle damages grapevine by feeding on its leaves, thereby limiting the plant’s ability to photosynthesize and, in turn, affecting grape quality.

**Table 1:** Overview of additional private management costs potentially caused by a Japanese beetle infestation.

Type of additional costs	Reason
Labor	Visual monitoring
	Physical removal of Japanese beetles on vine plants
	Spraying of plant protection products
Insecticides	Reduction of Japanese beetle population on vine plants
Mass/Pheromone traps^1^	Reduction of Japanese beetle population on vine plants

1 Mass/pheromone traps might be applied by the farmer to reduce population, however it should be noted that generally they are not recommended by phytosanitary experts as they might attract more Japanese beetles than they are able to capture (17).

### 2.2 Analysis

Our analysis was conducted in two parts. First, we provide a descriptive analysis of farmers’ perceptions of the beetle’s impact. Second, we use a partial budgeting approach to assess the beetle’s impact on private management costs.

The partial budget (PB) analysis is a commonly used method to assess the direct economic consequence of a change at the farm business level like the introduction of a pest (25, 26). It is one of several main economic impact assessment techniques used in pest risk analysis (26, 27), which focuses on the direct economic impact on the farmer. Other methods like the partial equilibrium modelling, input-output analysis and computable general equilibrium model also take into account indirect effects caused through price effects and linkages to other agricultural markets or sectors (26). However, depending on the purpose of the analysis and the contextual framework these methods might not be suitable (26). In our case, where the Japanese beetle invasion in Europe is still at an early phase affecting mostly grapevine farmers in the Piedmont region, we can assume that it has resulted only in limited indirect effects. Hence, the PB technique is the optimal choice because it focuses on the producer. The method is based on the notion that changes will lead to adjustments in the farming system such as reductions or increases in some costs and revenues (14, 25, 28). Adding up the negative and/or positive cost changes will lead to the overall net change in profit (26) as presented in **Table 2**. A positive net economic effect of a change can be achieved if the sum of the positive economic effects exceeds that of the negative effects. Thus, PB is a basic but popular tool due to its simplicity and transparency (26). However, it is not designed to analyze the profit or loss of a business as a whole but instead focuses only on the specific costs or revenue components that could be affected by the change (25, 26).

**Table 2:** Partial budgeting format. Based on the layout of Frem et al. (28) & Soliman et al. (26).

Costs		EUR	Benefits		EUR
**A)**	Additional costs		**C)**	Additional revenues	
	Control & protection costs				
	Labor costs				
**B)**	Reduced revenues		**D)**	Reduced costs	
	Yield and/or Quality losses				
**Total costs: A + B**			**Total benefits: C + D**		
**Net change in profit: C + D - A - B**					

This study assumes that a Japanese beetle infestation may potentially lead to a net decrease in farm income of grape producers. Our analysis focuses on comparing the economic consequences of a Japanese beetle infestation in a vineyard. To assess the per hectare net change in profit between affected and unaffected vineyard plots, the potential yield loss and associated costs per hectare of labor, insecticides and usage of other control methods like pheromone traps have been calculated. This calculation is based on primary and secondary data. To estimate the costs of labor, we used the wages established in the collective agreement for agriculture (18). The costs of control methods are based on average application rates of the farmers combined with recent market prices of the products (19–22).

## 3 Results

### 3.1 Sample characteristics

**Figure 2:**
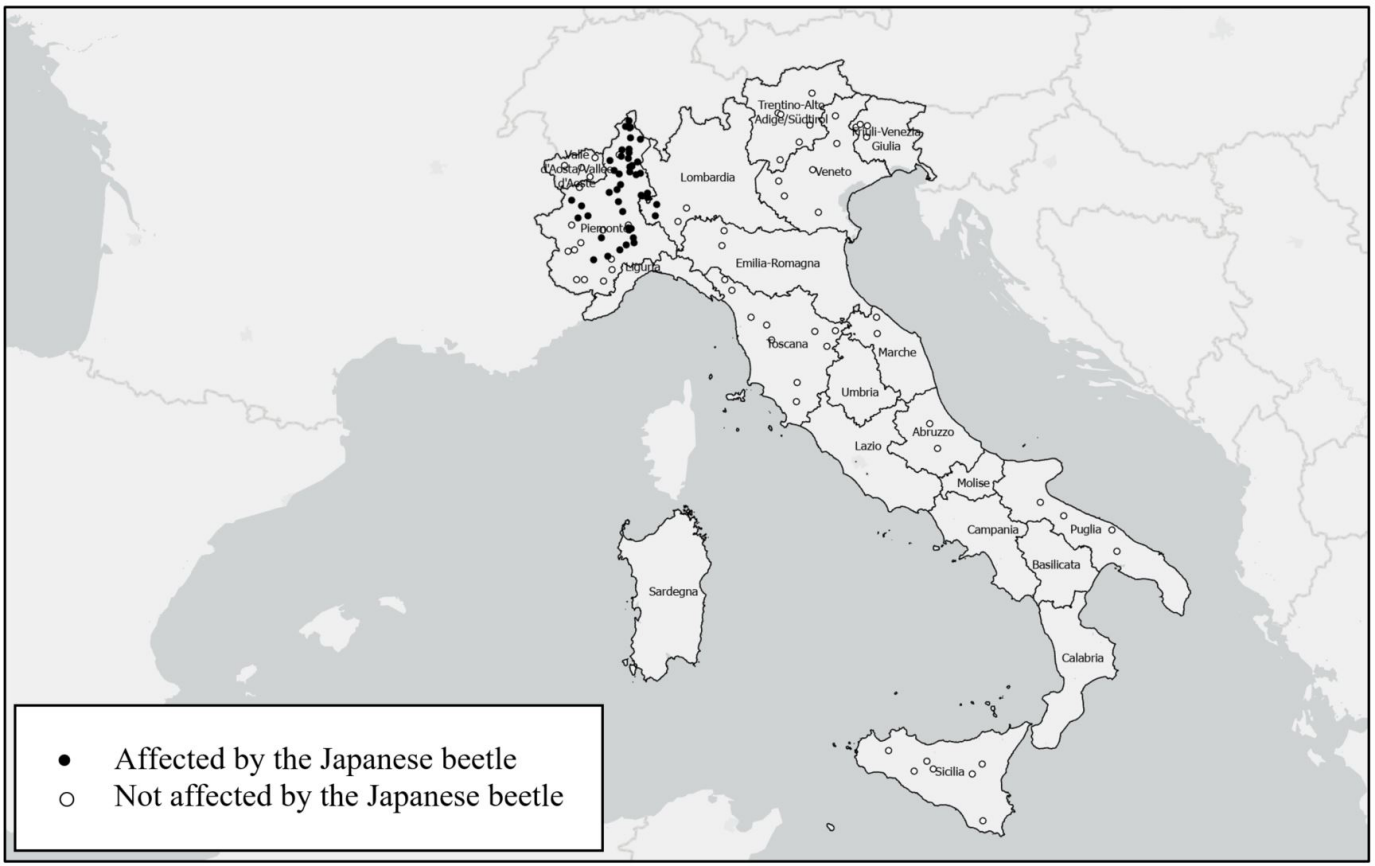
Overview of vineyard plot locations. Each dot represents a vineyard. The observations were randomly placed within the regions and do not represent the actual locations to preserve the anonymity of the survey participants.

A total of 65 Italian grape producers completed the survey. The respondents provided information on 118 vineyards plots, of which 47 were affected by the Japanese beetle in 2021 (**Figure 2**). Most of the affected vineyards are located in the Piedmont region.

The average age of the surveyed farmers was between 44 and 49 years. Over 90% of respondents had completed an upper secondary school education. In total, 27 farmers reported that they were affected by the beetle and 38 were not affected.

Most of the farmers affected by the beetle had an income below 10000 € per year and managed their farm conventionally as a side-business with a viticulture area of less than 10 hectares. In comparison, most unaffected farmers identified farming as their main business with over 50% having an annual income above 10000 € per year. Furthermore, almost 50% of unaffected producers were using organic standards of production and even though the viticulture area of most of the farmers is below 10 hectares, the sample includes a few big grape producing businesses with a viticulture area of more than 50 hectares and an annual income over 120000 € (see **Table 3**).

**Table 3:** Socioeconomic characteristics of the farmers in the sample.

Characteristics	Categories	Affected Farmers (N=27)			SD	Unaffected Farmers (N=38)			SD
		Respondents				Respondents			
		No.	(%)	Mean		No.	(%)	Mean	
Age				48.67	13.9			43.68	10.8
	>= 55 years	9	33.3			7	18.9		
Education	>= upper secondary	25	92.6			38	100		
Farm as side-business		12	70.6			9	26.5		
Total farm size (ha)				4.66	6.11			41.91	97
	< 10 ha	21	77.8			17	44.7		
	10-49.9 ha	6	22.2			16	42.1		
	> 50 ha	0	0			5	13.2		
Viticulture area (ha)				2.64	5.4			22	57.65
	< 10 ha	25	92.6			24	63.2		
	10-49.9 ha	2	7.4			11	28.9		
	> 50 ha	0	0			3	7.9		
Labor (non-paid/own, family)				1.75	0.85			4.62	5.01
Labor (paid, all season)				0.11	0.32			2.94	4.76
Labor (paid, seasonal)				0.4	0.75			6.17	9.44
Organic		3	11.1			18	47.4		
Farm income (€)	< 10.000	17	85			15	48.4		
	10.001-120.000	3	15			12	38.7		
	>120.000	0	0			4	12.9		

Due to missing observations, the respective percentage of respondents for the farm income variable refers to a total of 20 affected and 31 unaffected farmers.

### 3.2 Farmers’ perception of the Japanese beetle infestation

The assessment of farmers’ perception of the Japanese beetle infestation is based on the following four variables: the beetle’s impact on yield damage, quality damage, management costs and the possibility of having to stop the cultivation of grapevine on their vineyard due to the pest. For the first three variables, farmers could choose if they perceive the damage or the potential increase in management costs on their vineyard as negligible, minor, moderate, major or severe. For the fourth question, they could indicate the likelihood of having to stop grapevine cultivation on their vineyard due to the beetle as very unlikely, unlikely, neutral, likely or very likely. In total, up to 56 farmers responded to these perception questions for 103 vineyard plots. Fisher’s exact test was used to find out if there was a difference regarding perceptions of the Japanese beetle’s impact between affected and unaffected vineyard plots. The test indicated no statistically significant difference between affected and unaffected plots regarding the perception on yield (*p*=0.069), quality damage (*p*=0.796), management costs (*p*=0.479) and the possibility to stop the cultivation of grapevine on their plot (*p*=0.581).

**Figure 3:**
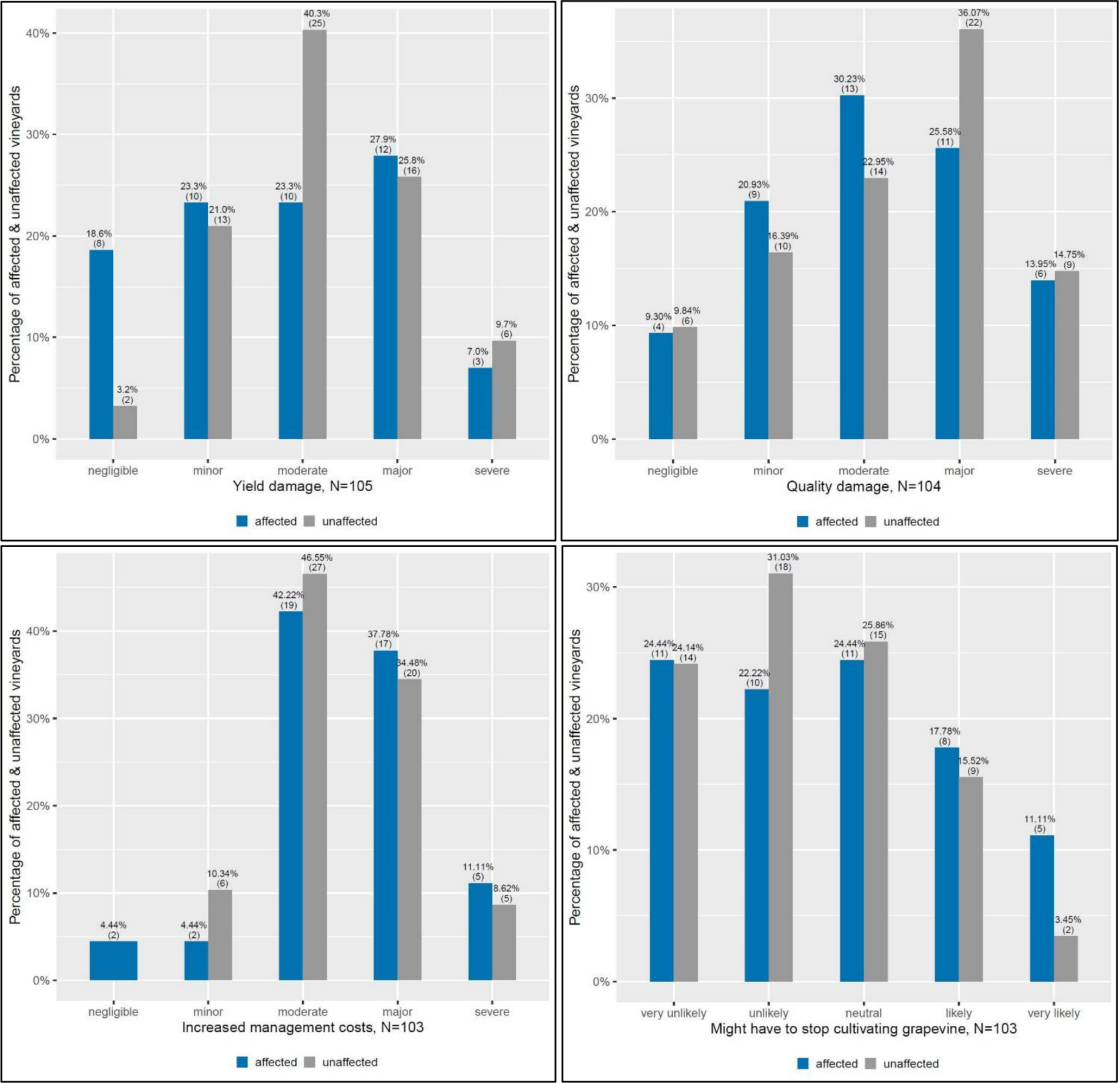
Farmer perceptions of how the spread of the Japanese beetle will affect future grapevine production.

This study found that for around 75% of the unaffected plots (N=45), farmers expect moderate to severe yield damage in the case of a further spread of the Japanese beetle (see **Figure 3**). For affected plots on the other hand, farmers expect that the beetle will lead to such yield damage levels for around 58% of the plots (N=25). For the remaining 42% of affected plots (N=18), producers state that a future spread of the beetle could also result in minor or negligible damage to their yield. Farmers’ perception of the beetle’s impact on grape quality shows that for around 70% of affected (N=30) and 73% of unaffected plots (N=43), producers believe in moderate up to severe quality damage. Farmers also expect moderate to severe increases in management costs due to the Japanese beetle for around 91% of affected (N=41) and 89% of unaffected plots (N=50). Only for around 9% of affected (N=4) and 11% (N=6) of unaffected plots it was indicated that a minor or negligible increase in management costs could be possible. Even though most of the farmers expect the Japanese beetle to increase their management costs, they think it is unlikely to very unlikely for around 47% of affected (N=4) and 57% of unaffected plots (N=6) that they will have to stop the cultivation of grapevine on their vineyard plot because of the pest. For around 29% of affected (N=13) and 16% of unaffected plots (N=9) however, producers indicated that it is likely to very likely.

In addition to evaluating farmers’ perception of the Japanese beetle impact on their grapevine production, we also wanted to find out how they assess the leaf damage tolerance of their vines. In order to do so, we asked the farmers for each of their plot(s) how much leaf damage they think that the vines can tolerate before experiencing significant negative impact on their grape yield and quality. In total 54 farmers gave an answer and provided estimates for 94 vineyard plots. Similarly as before, Fisher’s exact test was performed to explore differences in answers between affected and unaffected plots. The test revealed a statistically significant difference between affected and unaffected plots on the perception of vines’ leaf damage tolerance (*p*<0.001).

**Figure 4:**
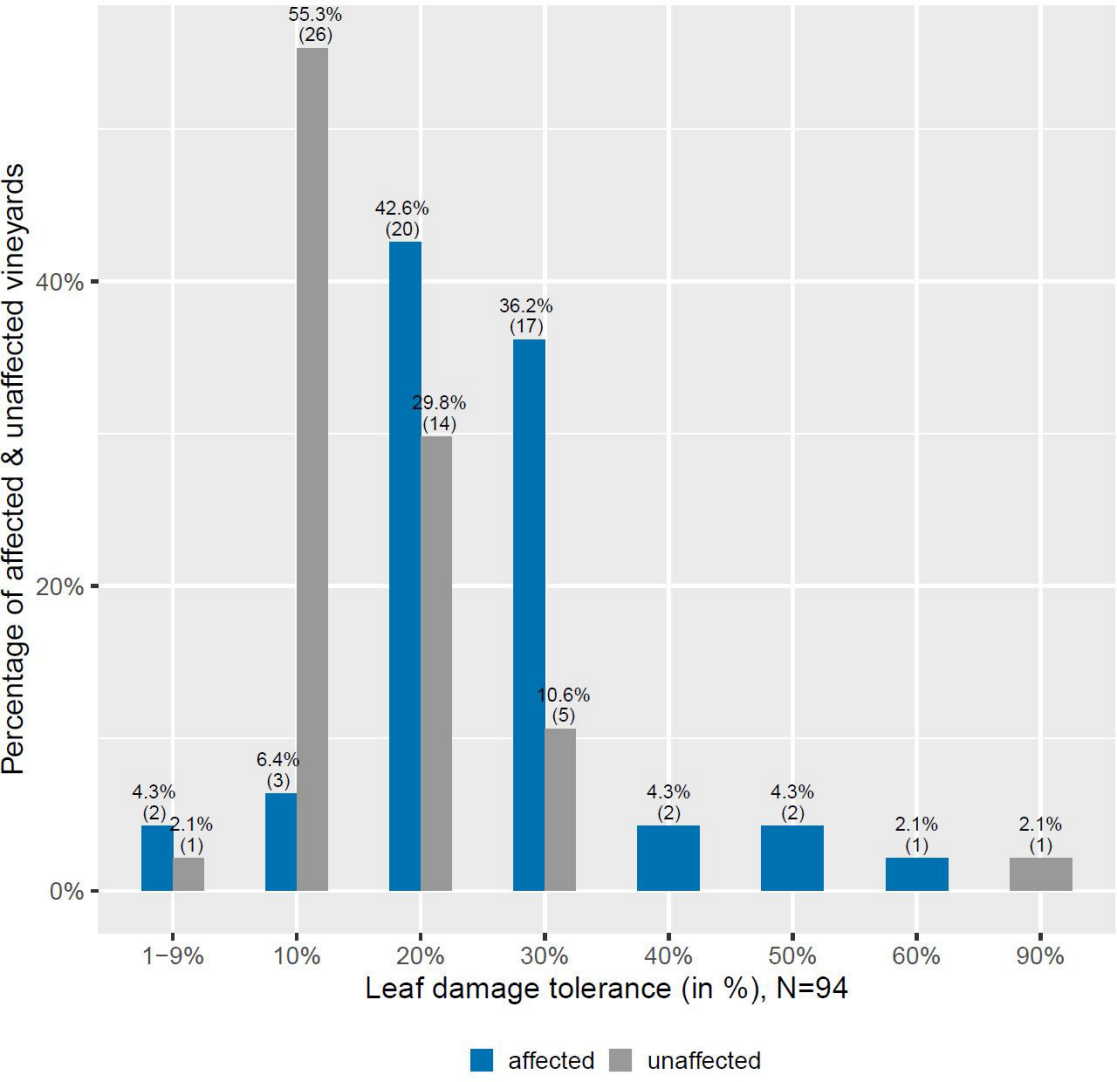
Farmer perception of the leaf damage tolerated until there is a significant negative impact on grape yield & quality.

According to the results of the survey, for the majority of unaffected plots (55%, N=26) farmers believe that their plants can only tolerate leaf damage levels up to 10% before experiencing a significant negative impact on their grape yield and quality (see **Figure 4**). However, for most of the affected plots (79%, N=37) farmers indicated a higher tolerance level, which is between 20 to 30%.

### 3.3 Partial budgeting analysis

To investigate the Japanese beetle’s impact on the private management costs of grape producers, we have calculated the average difference in yield, labor and control measures between affected and unaffected plots (see **Table 4**). Since most of the affected plots were managed conventionally, we decided to exclude organically managed vineyards from the sample in order to control for potential differences in management practices. The sample for the PB analysis hence includes 42 affected and 38 unaffected plots.

**Table 4:** Overview of yield, labor and control measures of affected and unaffected vineyard plots.

Particulars	Affected Plot (N=42)		Mean application no. (if indicated)	Cost (€ per application & ha)	Unaffected Plot (N=38)		Mean application no. (if indicated)	Cost (€ per application & ha)
	(%)	Mean			(%)	Mean		
Yield per ha (in quintals)	71.4	50.4			71.1	62.9		
Price (€ per quintal)		77.3				77.3		
Labor per ha (in days)	90.5	147.5			55.3	116.4		
Labor costs (€/hour)		6.9				6.9		
Insecticides								
Acetamiprid	81.0		2.1	66.3	48.5		1.5	66.3
Clorantraniliprolo	4.8		*1.0*	59.7	0.0		0.0	59.7
Deltamethryn	45.2		2.1	25.6	3.0		2.0	25.6
Emamectina benzoato	0.0		0.0	52.8	6.1		1.0	52.8
Etofenprox	9.5		1.0	54.4	27.3		1.0	54.4
Flupyradifuron	4.8		2.0	38.8	24.2		1.0	38.8
Metossifenozide	0.0		0.0	39.2	6.1		1.0	39.2
Tau-fluvalinate	0.0		0.0	23.9	6.1		*1.0*	23.9
Bacillus thuringiensis	0.0		0.0	28.8	12.1		2.0	28.8
Beauveria bassiana	4.8		*1.0*	133.8	0.0		0.0	133.8
Insect soap	0.0		0.0	2.4	6.1		*1.0*	2.4
Orange oil	2.4		2.7	52.5	9.1		3.1	52.5
Paraffin oil	0.0		0.0	176.0	6.1		*1.0*	176.0
Pyrethrine	2.4		2.0	92.4	15.2		1.0	92.4
Rapeseed oil	2.4		2.0	103.2	3.0		*1.0*	103.2
Spinosad	0.0		0.0	95.4	15.2		1.0	95.4
Other control methods								
Mass/pheromone trap (trap per ha)	16.7		15.4	10.3	6.1		2.0	10.3
Insect picking by hand (h per ha)	35.7		38.7		0.0		NA	
Visual inspection (h per ha)	54.8		20.6		51.5		2.4	

Values written in italics indicate that the product was applied but the corresponding information on the number of applications is missing and therefore one application was assumed. The per hectare costs of insecticides and other control methods is based on the recommended dosage per hectare combined with mean market prices of the products containing the active ingredient sourced from online market shops (19–22). The costs of labor are based on the wages established in the collective agreement for agriculture (18). For the calculation of the mean yield, entries of 0 and more than 200 quintals per ha were removed. Similarly labour per ha of over 800 days and a price of less than five Euros per quintal were unlikely and were therefore removed from the dataset.

We combined the average yield per hectare of affected and unaffected plots with the indicated average price of conventional farmers, which is 77 € per quintal. The price estimate seems to be realistic when compared to the latest available producer price of grapes in Italy from Eurostat, which is 66.2 € per quintal for the year 2018 (29). In addition, we asked the participants about the total amount of labor, which was necessary to manage their vineyard plot in 2021. While it took on average 116 days to manage one hectare when not affected by the Japanese beetle, this increases to around 148 days when affected by the pest. From **Table 4** we can see that the difference in labor of affected plots can be explained through an increase in other control methods like visually inspecting the vineyard or manually picking the beetle, which are labor-intensive control measures. It should be noted that we did not include these two control measures in the PB analysis as those costs are already accounted for in the labor per hectare estimate and are shown only to provide additional information on the labor differences. The difference in costs of control measures per hectare have been calculated taking into account the average difference in application rates between affected and unaffected plots and multiplying it with the share of plots that use the specific product. This value is then combined with recent market prices of the products (19–22). One of the main differences in the use of control products is that a higher percentage of affected plots was treated with insecticides containing the active ingredient acetamiprid (80%) and deltamethryn (45%) (see **Table 4**). In addition, the average application rate for products with acetamiprid was higher for affected plots. This increase in application for insecticides containing acetamiprid and deltamethryn makes sense since they are among the active substances registered against adults of the Japanese beetle (3).

**Table 5** shows the results of the PB analysis based on the above- mentioned cost estimates. We found that vineyards experience, on average, a reduction in net income of around 2727 € per infested hectare compared to unaffected ones. Among the different cost components, labor was the main additional expense affecting the net income loss followed by a revenue reduction of around 966 € due to yield loss. The cost difference in the usage of control methods is around 47 € per infested hectare.

**Table 5:** Partial budget analysis and net change in profits of Japanese beetle affected vineyard compared to unaffected vineyard.

Affected vs. Unaffected						
Costs		€ EUR/ha		Benefits		€ EUR/ha
A)	Additional costs			C)	Additional revenues	0
	Insecticides	21.62				
	Other Control methods	25.16				
	Labor	1,715.06				
B)	Reduced revenues			D)	Reduced costs	0
	Yield	965.60				
**Total costs: A + B**		2,727.44		**Total benefits: C + D**		0
**Net change in profit: C + D - A – B**			-2,727.44			

## 4 Discussion

In this study, we assess two sides of the Japanese beetle infestation’s impact in Italy through farmers’ perceptions and the partial budget analysis.

In our investigation of farmers’ perception of the effect of the Japanese beetle on grapevines, we find that farmers expect at least moderate yield and quality damages for the majority of plots (58-76%, N=25-47). While farmers consider the possibility that they might have to stop their grapevine cultivation as (very) unlikely or neutral (71-81%, N=32-47), at least moderately increased management costs are expected for the majority of vineyard plots (90-91%, N=41-52). However, even though not statistically different at the 95% level, there are some differences in opinion for affected and unaffected plots, which are worth mentioning. Although unaffected farmers are concerned about future yield damage, a large percentage of affected farmers (42%, N=18) are less afraid about the beetle’s impact on yield. This could be due to the fact that in our sample most of the affected producers have smaller farms, which they manage conventionally as a side-business. The group of the unaffected farmers, on the other hand, may be more concerned about the future spread of the beetle as most of them run their farms full-time and almost 50% of them are producing with organic standards, for which only limited control options exist at the moment.

The responses of farmers regarding the leaf damage tolerance of vines also shows differences between affected and unaffected plots, which are statistically significant. While for 55% of the unaffected plots (N=26), farmers state that their plants can only tolerate leaf damage up to 10% before experiencing a significant negative impact on their yield and quality, they do so only for 6.5% of the affected plots (N=3). For around 90% of plots affected by the beetle (N=42) higher tolerance levels have been indicated with most of them stating that a defoliation between 20 to 30% is possible. As farmers with affected plots differ in terms of perceived leaf damage tolerance, farmers with affected plots might have more knowledge and experience with leaf damages and accurately anticipate the impact of defoliation on their plants. The expectations of these experienced farmers are in line with a recent study from the US, which found that with a defoliation higher than 30 to 35%, the feeding of the Japanese beetle starts to show negative effects on various quality parameters of the wine grape variety “Frontenac” (30).

In our partial budgeting analysis, we find that vineyards affected by the Japanese beetle experience, on average, a net loss of around 2727 € per infested hectare with labor being one of the key cost increases. Additional labor may be necessary due to increased intensive monitoring, additional applications of insecticides or even manual picking of the adult beetles every morning during their flight period. Another important cost factor is the decrease in revenue due to yield loss. Hence, Japanese beetle affected vineyard plots have a net decrease in income per infested hectare due to yield loss, additional applications of insecticides and increased workload. However, the observed variations between affected and unaffected vineyards cannot be interpreted as causal. As weather conditions and other pest outbreaks could play a major role, future studies should control for these factors. In addition, the PB analysis relies on a small number of observations and the descriptive results can therefore not be seen as representative of Italy.

In summary, the Japanese beetle can negatively affect viticulture in Italy through a potential yield loss and increased labor and control costs. This is also reflected in the perception of the farmers. One of the main cost components identified in this study is the additional labor required to deal with the pest. Farmers may be forced to handpick the adult beetles from their vines because only few control methods against the beetle currently exist. While spraying of insecticides only helps in the short term, re-infestation of the vines is possible within a few days after treatment (17). Hence, additional insecticide applications are necessary but not unlimited due to their negative effects on beneficial organisms and the environment. Additionally, increased consumer concerns about products with pesticide residues as well as potential problems with pests developing resistance to synthetic chemical products (31) make future research and investment in the development of effective and environmentally friendly control measures necessary. Finally, our study tries to give a first picture on how the Japanese beetle affects grapevine producers in Italy. However, as the beetle is spreading, future studies at the farm level are essential, especially due to our small sample size and since the affected producers in our sample represent mostly small farms. In addition, also the impact of the beetle on specific grape varieties as well as other host crops like maize, blueberry or hazelnut, at different levels of infestation across Europe needs to be evaluated.

## Data availability statement

The raw, anonymized data supporting the conclusions of this article are not available as the authors are not entitled to disclose the data to third parties or institutions outside the IPM Popillia research group. Requests to access the datasets should be directed to Franziska Straubinger, franziska.straubinger@tum.de..

## Ethics statement

Ethical review and approval was not required for the study on human participants in accordance with the local legislation and institutional requirements. The patients/participants provided their written informed consent to participate in this study.

## Author contributions

FS and TV: conceptualization and methodology. FS: formal analysis and writing of original draft. TV and BE: reviewing. BE and JS: resources, project administration, and funding acquisition. All authors provided a final review and approved the submitted version.

## Footnotes

1 Notification from central phytosanitary service - Italy to the europhyt portal outbreak. (2023).
