# Supplementary material for: Private management costs of Popillia japonica: a study of viticulture in Italy
Source: Front Insect Sci. 2023 Jun 14;3:1176405. doi: 10.3389/finsc.2023.1176405 (PMC10926395; doi:10.3389/finsc.2023.1176405)
Supplement: Supplementary file 1 [file DataSheet_1.pdf]

frontiers

Supplementary Material

Supplementary material A: Extracts from the field survey questionnaire

In the following section, you will be asked questions about your company. We collect this information in order to be able to compare Japanese beetle affected and not-affected farmers and to find out about i.e. potential yield reduction, increased labour use, etc. If you do not wish to answer a particular question, you can skip every question. However, for our analysis it is important that you answer all questions as completely as possible.

Unless otherwise stated, please refer to the last fiscal year 2020/2021 or the calendar year 2021, respectively in your answers.

In which year were you born? _____

Please, indicate your highest education level

□ no formal qualification □ university degree (bachelor) □ not specified

□ primary education □ university degree (master)

□ lower secondary education □ university degree (PhD)

□ upper secondary education □ other (please specify)

Is your company a ...

□ Main holding (no or only little/limited off-farm income)

□ Side business (off-farm income higher than farm income)

□ Not specified

Please indicate approximately your average imputed annual farm profit (= profit after remuneration of own labour and invested equity capital)

□ < 1.000 EUR □ 20.001-40.000 EUR □ 100.001-120.000 EUR

□ 1.000-5.000 EUR □ 40.001-60.000 EUR □ >120.001 EUR

□ 5.001-10.000 EUR □ 60.001-80.000 EUR □ Not specified

□ 10.001-20.000 EUR □ 80.001-100.000 EUR

Please indicate your total agricultural area under production in 2021 in hectares (ha). _____

Please indicate the agricultural area used for grape production in 2021 in hectares (ha). _____

How many different grapevine plots did you cultivate in 2021?

- 1
- 2
- 3
- 4
- more than 4 (please specify)

Are you solely cultivating grapevine?

- yes
- no

Please indicate your other farm activities apart from viticulture

□ other permanent crops	□ livestock
□ arable crops	□ other
□ horticulture	□ not specified

In the following section, please provide information for 2 of your vineyards. We suggest you choose the two largest vineyards affected by the Japanese beetle in 2021. If this is not possible, refer to the vineyards that have been affected in recent years or, in the event that your vineyards have not been affected at all, to your two largest vineyards.

Please note: as before, different grapevine plots here mean: plots, which are geographically located apart from each other (i.e. located in different areas or separated from each other by a road, field, etc.)

Where are your vineyard plot(s) located?
Please indicate the applicable zip code. We assure you that we will treat your information confidentially. Results are published only in highly aggregated form.

Plot 1 _____

Plot 2 _____

What is the area of your vineyard plot (in ha)?

Plot 1_____

Plot 2 _____

Please provide some general information on your vineyard plots

Ownership | Slope steepness | Soil fertility | Mechanization | PDO/PGI or variety indication | Age of vines

Plot 1

Plot 2

How many days of work were approximately necessary to manage your plots in 2021?
Please indicate approx. the total number of days (1 day = 8 hours) that you, family members or external staff worked on your plots in 2021. For example: seasonal worker employed for 6 months on plot 1 (=20 work days per month), means 120 days in total for the seasonal worker and plot 1.

Plot 1 _____

Plot 2 _____

Were your plots affected by the Japanese beetle in 2021?

	Yes	No	Not specified
Plot 1	□	□	□
Plot 2	□	□	□

How do you think will the Japanese beetle spread affect your grapevine production in the future? Please indicate how the Japanese beetle will affect your yield, grape quality, management costs and overall grapevine production.

Yield Damage | Quality Damage | Increased Management Costs | Might have to stop cultivating grapevine

Plot 1

Plot 2

What percentage of leaf damage do you think your plants can tolerate without experiencing significant negative impact on your grape yield & quality?
Please answer this question whether or not you have been affected by the Japanese beetle.

	1-9%	10%	20%	30%	40%	50%	60%	70%	80%	90%	95%
Plot 1	□	□	□	□	□	□	□	□	□	□	□
Plot 2	□	□	□	□	□	□	□	□	□	□	□

What was your average yield in 2021 (in quintals)?

Plot 1 _____

Plot 2 _____

Have you already been paid for your harvest in 2021?

	Yes, fully paid	Partially payed	Not paid at all yet	I do not sell my grapes because I process them myself
Plot 1	□	□	□	□
Plot 2	□	□	□	□

What was the average price per quintal in 2021 (EUR/quintal)?

Plot 1 _____

Plot 2 _____

What was the average price per quintal for the already paid harvest of 2021 (in EUR/quintal)?

Plot 1 _____

Plot 2 _____

What price per quintal do you expect to get for the rest of your harvest from 2021 (EUR/quintal)?

Plot 1 _____

Plot 2 _____

What price per quintal do you expect to get for your harvest in 2021 (EUR/quintal)?

Plot 1 _____

Plot 2 _____

In the following, please provide some information on your current plant protection practices against insect pests in general. Please refer to the same 2 plots as in the previous section.

Which plant protection methods did you use on plot 1 against insect pests in 2021?

Chemical insecticides	*Biological insecticides*	*Monitoring*	*Mechanical control*
□Abamectin	□Azadirachtin	□Visual inspection	□Mass trapping
□Acetamiprid	□Bacillus t. sub. aizawai	□Yellow traps	□Vibrational mating disruption
□Cypermethrin	□Bacillus t. sub. kurstaki	□other monitoring (please specify)	□Insect picking by hand
□Clorantraniliprole	□Beauveria bassiana		□Nets
□Dazomet	□Metarhizium anisopliae var. anisopliae		□Other mechanical control (please specify)
□Deltamethryn	□Pyrethrine		
□Emamectina benzoato	□Insecticidal soaps		

□Esfenvalerate	□Paraffin oil
□Etofenprox	□Spinosad
□Flupyradifurone	□Rapeseed oil
□Lambda-Cyhalothrin	□Other biological active ingredient (please specify)
□Metam-potassium	
□Metam-sodium	
□Metoxyfenozide	
□Pyriproxyfen	
□Spinetoram	
□Spirotetramat	
□Sulfoxaflor	
□Tau-fluvalinate	
□Tebufenozide	
□Other chemical active ingredient (please specify)	

Please provide some information on your used control methods on plot 1

	Number of applications during production cycle	Number of nets/traps or hours
List of chosen PPPs		

Did you use the same insect pest control methods on plot 2?

- yes - the insect pest management of both plots is completely the same
- partially different - the insect pest management is slightly different between both plots
- no - the insect pest management of both plots differs
- not specified

Which plant protection methods did you use on plot 2 against insect pests in 2021?

Chemical insecticides	*Biological insecticides*	*Monitoring*	*Mechanical control*
□Abamectin	□Azadirachtin	□Visual inspection	□Mass trapping
□Acetamiprid	□Bacillus t. sub. aizawai	□Yellow traps	□Vibrational mating disruption

□Cypermethrin	□Bacillus t. sub. kurstaki	□other monitoring (please specify)	□Insect picking by hand
□Clorantraniliprole	□Beauveria bassiana		□Nets
□Dazomet	□Metarhizium anisopliae var. anisopliae		□Other mechanical control (please specify)
□Deltamethryn	□Pyrethrine		
□Emamectina benzoato	□Insecticidal soaps		
□Esfenvalerate	□Paraffin oil		
□Etofenprox	□Spinosad		
□Flupyradifurone	□Rapeseed oil		
□Lambda-Cyhalothrin	□Other biological active ingredient (please specify)		
□Metam-potassium			
□Metam-sodium			
□Metoxyfenozide			
□Pyriproxyfen			
□Spinetoram			
□Spirotetramat			
□Sulfoxaflor			
□Tau-fluvalinate			
□Tebufenozide			
□Other chemical active ingredient (please specify)			

Please provide some information on your used insecticides on plot 2

	Number of applications during production cycle	Number of nets/traps or hours
List of chosen PPPs		
